# Characteristics and Transmission Dynamics of Global Travel-Related Mpox Cases Caused by Clade Ib Monkeypox Virus

**DOI:** 10.3201/eid3202.251530

**Published:** 2026-02

**Authors:** Henry Laurenson-Schafer, Martina McMenamin, Lennox Kesington Ebbarnezh, Ioannis Karagiannis, Viema Biaukula, Rosamund Lewis, Masaya Kato, Michel Muteba, Jeremias D. Naiene, Felix Sanni, Tshewang Dorji, Lorenzo Subissi, Marc-Alain Widdowson, WHO Mpox Collaborative Surveillance Group, Ana Hoxha, Olivier le Polain de Waroux

**Affiliations:** World Health Organization (WHO) Health Emergencies Programme, Geneva, Switzerland (H. Laurenson-Schafer, M. McMenamin, L.K. Ebbarnezh, R. Lewis, L. Subissi, A. Hoxha, O. le Polain de Waroux); WHO Health Emergencies Programme, Regional Office for the European Region, Copenhagen, Denmark (I. Karagiannis, M.-A. Widdowson); WHO Health Emergencies Programme, Regional Office for the Western Pacific Region, Manila, Philippines (V. Biaukula); WHO Health Emergencies Programme, Regional Office for the South-East Asian Region, New Delhi, India (M. Kato); WHO Health Emergencies Programme, Regional Office for Africa, Brazzaville, Republic of the Congo (M. Muteba, F. Sanni); WHO Health Emergencies Programme, Regional Office for the Eastern Mediterranean Region, Cairo, Egypt (J.D. Naiene); WHO Health Emergencies Programme, Regional Office for the Americas, Washington, DC, USA (T. Dorji)

**Keywords:** Mpox, monkeypox virus, viruses, zoonoses, sexually transmitted infections, clade Ib, imported cases, PHEIC

## Abstract

We examined 89 travel-related clade Ib monkeypox virus cases detected in 33 countries during August 2024–July 2025. Most cases were men; about one third led to secondary transmission. Secondary transmission risk was highest among sexual, then household, contacts. Those groups should be the focus of response strategies and interventions.

In September 2023, an outbreak of a new strain of monkeypox virus (MPXV) emerged in the nonendemic Sud-Kivu province of the Democratic Republic of the Congo (DRC); the outbreak was driven by sustained human-to-human transmission (*1*). Mpox cases previously reported in the DRC described outbreaks linked to zoonotic spillover in forested areas (*2*). The sustained human-to-human transmission in the 2023 outbreak led to proposed designation of a new MPXV subclade, clade Ib (*1*). Subsequent spread to countries neighboring the DRC (*3*) prompted the World Health Organization (WHO) to declare a second public health emergency of international concern for mpox on August 14, 2024 (*4*). We collated data from travel-related cases and their contacts reported to WHO or published separately to describe transmission dynamics, estimate secondary attack rates (SAR), and help define risk factors for clade Ib MPXV infection.

## The Study

By June 19, 2025, MPXV clade Ib had been reported from 14 countries in Africa. Travel-related cases, as defined by WHO (*5*), were reported from 19 countries outside of Africa and led to secondary cases in 8 of the destination countries. 

**Figure 1:**
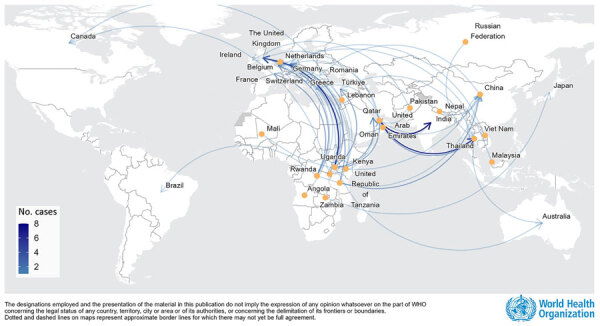
Transmission dynamics among global travel-related mpox cases caused by clade Ib monkeypox virus. Yellow dots represent country of exposure; arrows represent direction of travel for mpox cases reported as of June 19, 2025. Some of the cases included in the article are not shown because the exact country of exposure was not known. Data source: World Health Organization (WHO); produced by WHO Health Emergencies Programme; copyright WHO 2026, all rights reserved.

We used case data that was shared with WHO under the provisions of the International Health Regulations (2005) (*6*) or published separately. We collated data from 127 mpox cases, 124 confirmed and 3 suspected; 89 were travel-related, 34 were secondary, and 4 were unlinked. Of the 89 travel-related cases, 70 case-patients reported having been exposed in Africa and 18 outside Africa; 1 had unknown exposure origin (Figure 1). All imported cases were among adults, except 1 of unknown age (missing data); 88 cases had data on sex available, 67 (76%) were men and 21 (24%) were women; 25 travel-related cases led to 34 secondary cases in the country of notification, including cases among 6 men, 19 women, and 6 children (<17 years of age). Among secondary cases, 15/25 (60%) adults reported sexual contact or likely sexual contact between the index case (i.e., unspecified contact with the index case and partners).

**Figure 2:**
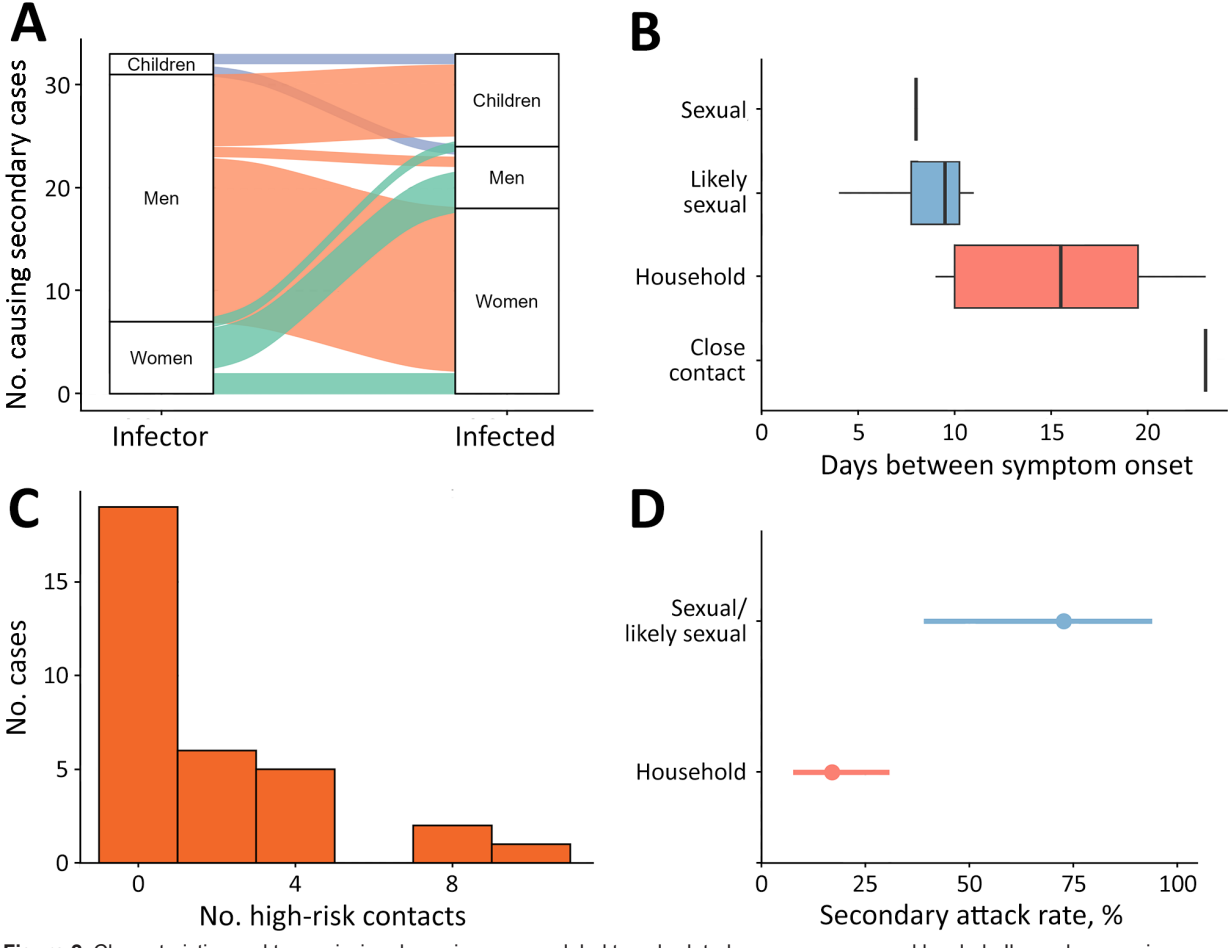
Characteristics and transmission dynamics among global travel-related mpox cases caused by clade Ib monkeypox virus. A) Sankey diagram of secondary transmission events among 33 transmission events and secondarily infected persons. Index cases causing multiple secondary cases were counted per secondary case. Children are persons <17 years of age. B) Box and whisker plot representing the serial interval for symptom onset by reported contact type for 14 case–contact pairs. Close contact is physical nonsexual interaction between the index and secondary case. Box left and right edges represent first and third quartiles, vertical lines indicate medians, and whiskers represent ranges. C) Number of high-risk contacts per each imported MPXV clade Ib case with available data among 33 high-risk contacts. D) Secondary attack rate (with 95% CIs) by reported contact type. Household contacts represent 8 secondary cases from 47 contacts; sexual contacts represent 8 secondary cases from 11 contacts.

Of the 33 secondary cases with data available for both index and secondary cases, most (73%) resulted from men, who transmitted MPXV to mostly women and children, followed by women (21%), who infected mostly men and other women. Secondarily infected children (6%) infected 1 child and 1 man (Figure 2, panel A). Using 14 case pairs, we estimated that the mean serial interval (SI) for infection was 12 (95% CI 4–27) days. We estimated that the SI for sexual and likely sexual contact was 9 (95% CI 5–14) days, which was shorter than the SI for nonsexual household contact, 15 (95% CI 8–29) days (Figure 2, panel B, raw data boxplots). That SI difference could partly be because of the shorter incubation period and higher infectious dose for sexual transmission (*7*; F.K. Kaiser et al., unpub. data., https://doi.org/10.1101/2025.08.14.669880).

Contact data were available from 50 cases, and we were able to disaggregate risk status of contacts for 32 of those cases. We defined high-risk contacts as household members, sexual contacts, or both. When no further information was available, we relied on reporting countries’ definitions of high-risk contacts. Using those data, we identified a total of 74 high-risk contacts from 33 cases, 1 of which did not have enough information available to disaggregate further into contact type (Figure 2, panel C). Of the 25 travel-related index cases from whom secondary transmission was reported, a median of 1 (range 1–4) secondary case occurred. We estimated the SAR for household contacts was 17% (95% CI 8%–31%) and for sexual contacts was 73% (95% CI 39%–94%) (Figure 2, panel D). No secondary cases were reported among community contacts or contacts in healthcare settings.

The first limitation of our analysis is the small sample size because information was missing for some cases. Second, the sexual contact SAR is primarily limited to transmission between spouses or partners, and the high estimate might reflect repeated sexual exposure, prolonged close contact within the household, or both, together with a possible underreporting of sexual contacts outside the household. Finally, travel-associated cases could be missed, especially in countries where mpox is strongly stigmatized, and our estimates might not be generalizable beyond the settings described.

## Conclusions

Our results highlight that close contact is a key driver of MPXV clade Ib outbreaks and show that sexual contact carried the highest transmission risk, followed by household contact. We found no evidence of transmission beyond settings involving close and prolonged contact in the available data. Other studies have also acknowledged the role of sexual contact as the most efficient MPXV transmission route (*8*,*9*). Of note, secondary household cases were identified during the MPXV clade Ib outbreak reported here, but during the 2022–23 clade IIb outbreak, such events were rare; before August 2024, WHO recorded only 15 (0.2%) of 7,794 mpox cases in children <15 years of age among those exposed in the household (data not shown). Whether that disparity reflects differences in household and social contact structures or intrinsic viral properties remains unclear. The higher risk associated with sexual exposure and household contact should inform response strategies and priority interventions for populations most at risk.
